# Crystal structure of dimethyl 4,4′-di­meth­oxy­biphenyl-3,3′-di­carboxyl­ate

**DOI:** 10.1107/S2056989016002449

**Published:** 2016-02-13

**Authors:** Fredrik Lundvall, Pascal D. C. Dietzel, Helmer Fjellvåg

**Affiliations:** a Centre for Materials Science and Nanotechnology, Department of Chemistry, University of Oslo, PO Box 1126, 0315 Oslo, Norway; b Department of Chemistry, University of Bergen, PO Box 7803, 5020 Bergen, Norway

**Keywords:** crystal structure, inter­mediate compound for organic linkers in MOF synthesis, centrosymmetric mol­ecule

## Abstract

In the title compound, C_18_H_18_O_6_, the benzene rings are coplanar due to the centrosymmetric nature of the mol­ecule, with an inversion centre located at the midpoint of the C—C bond between the two rings. Consequently, the methyl carboxyl­ate substituents are oriented in a *trans* fashion with regards to the bond between the benzene rings. The methyl carboxyl­ate and meth­oxy substituents are rotated slightly out of plane relative to their parent benzene rings, with dihedral and torsion angles of 18.52 (8) and −5.22 (15)°, respectively. The shortest O⋯H contact between neighbouring mol­ecules is about 2.5 Å. Although some structure-directing contributions from C—H⋯O hydrogen-bonding inter­actions are possible, the crystal packing seems primarily directed by weak van der Waals forces.

## Abstract 2

The title compound is an inter­mediate in the synthesis of linkers for coordination polymers. Centrosymmetric mol­ecules are packed along the *a* axis to form corrugated layers parallel to the *ac* plane.

## Chemical context   

The title compound is an inter­mediate in the synthesis of 4,4′-di­meth­oxy­biphenyl-3,3′-bi­phenyldi­carb­oxy­lic acid, an organic linker for use in the synthesis of coordination polymers (Lundvall *et al.*, 2016 ▸). The title compound, C_18_H_18_O_6_, has previously been reported (Wang *et al.*, 2009 ▸; Kar *et al.*, 2009 ▸), however, its crystal structure was undetermined up until now.
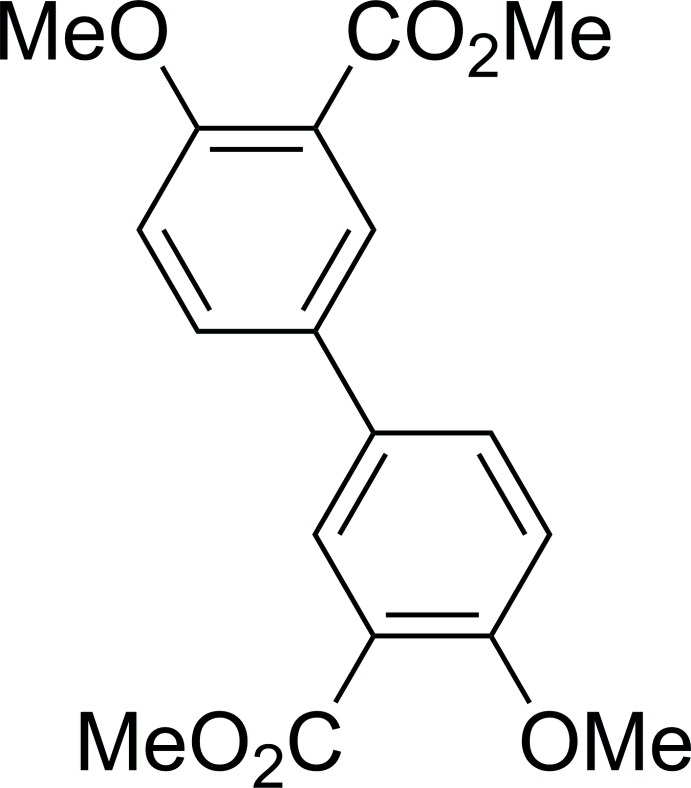


## Structural commentary   

**Figure 1:**
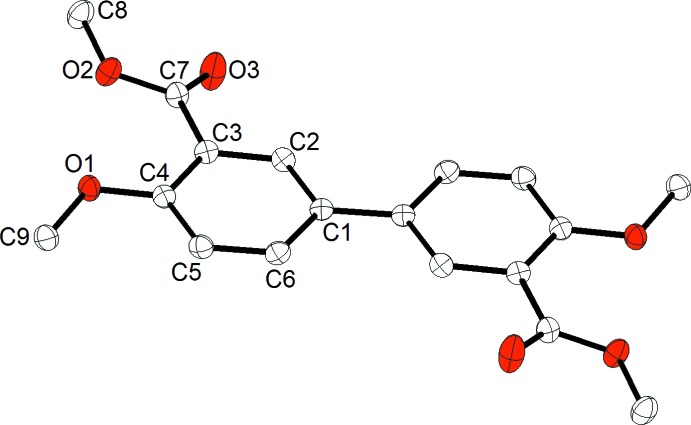
The mol­ecular structure of the title compound with atom labels and 50% probability displacement ellipsoids. Non-labelled atoms are generated by the symmetry code (−*x* + 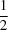, −*y* + 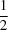, −*z*). H atoms have been omitted for clarity.

The asymmetric unit of the title compound comprises one half of the mol­ecule, with an inversion centre located at the midpoint of the benzene–benzene bond (Fig. 1 ▸). The benzene rings are coplanar due to symmetry. This is somewhat unexpected since a slight torsion between the two rings is a common feature in biphenyl compounds. The methyl carb­oxyl­ate substituents are oriented *trans* relative to the benzene–benzene bond, and the plane of the substituent makes a dihedral angle of 18.52 (8)° relative to the parent benzene ring. The meth­oxy substituent is nearly coplanar with the parent benzene ring, and a torsion angle C5—C4—O1—C9 of only −5.22 (15)° is observed. The methyl groups of the methyl carboxyl­ate and meth­oxy substituents are oriented away from each other to accommodate the steric demands of these groups.

## Supra­molecular features   

**Figure 2:**
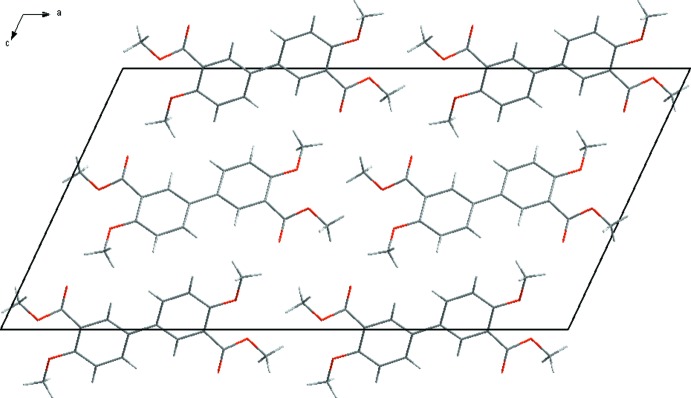
Packing diagram of the title compound viewed along the *b* axis.

**Figure 3:**
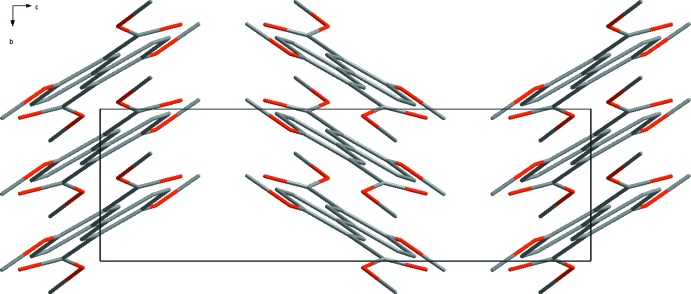
Packing diagram of the title compound viewed along the *a* axis. H atoms have been omitted for clarity.

**Figure 4:**
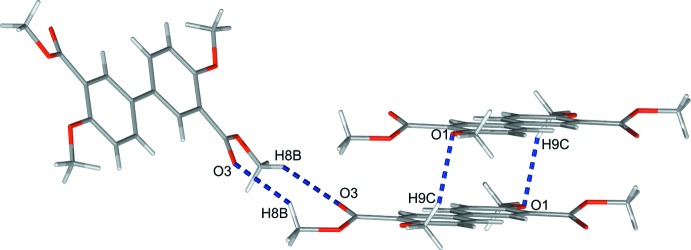
Graphical representation of the shortest inter­molecular O⋯H contacts, illustrated as dashed blue lines.

The mol­ecules are packed in the unit cell with the axis of the biphenyl scaffolds parallel to each other. The axis of the biphenyl moiety is oriented approximately 20° off the *a* axis of the unit cell (Fig. 2 ▸), and the mol­ecules form corrugated layers extending parallel to the *ac* plane (Fig. 3 ▸). The packing is not directed by strong inter­molecular bonding since the shortest O⋯H contact is about 2.5 Å (Table 1 ▸). However, weak C—H⋯O inter­actions between neighbouring mol­ecules seem to have an influence on the crystal packing (Fig. 4 ▸).

## Synthesis and crystallization   

The title compound was synthesized by a slightly modified procedure of the method described by Wang *et al.* (2009 ▸). Synthetic details are given in the Supporting Information of our recent contribution (Lundvall *et al.*, 2016 ▸). Single crystals suitable for structure determination were obtained by recrystallizing the title compound from chloro­form solution.

## Refinement   

Crystal data, data collection and structure refinement details are summarized in Table 2 ▸. H atoms were positioned geom­etrically at distances of 0.95 (CH) and 0.98 Å (CH_3_) and were refined using a riding model with *U*_iso_(H) = 1.2*U*_eq_(CH) and *U*_iso_(H)=1.5*U*_eq_(CH_3_).

## Supplementary Material

Crystal structure: contains datablock(s) I, New_Global_Publ_Block. DOI: 10.1107/S2056989016002449/wm5270sup1.cif

Structure factors: contains datablock(s) I. DOI: 10.1107/S2056989016002449/wm5270Isup2.hkl

Supporting information file. DOI: 10.1107/S2056989016002449/wm5270Isup3.cml

CCDC reference: 1452330

Additional supporting information:  crystallographic information; 3D view; checkCIF report

## Figures and Tables

**Table 1:** Hydrogen-bond geometry (Å, °)

*D*—H⋯*A*	*D*—H	H⋯*A*	*D*⋯*A*	*D*—H⋯*A*
C9—H9*C*⋯O1^i^	0.98	2.55	3.4759 (15)	158
C8—H8*B*⋯O3^ii^	0.98	2.50	3.3407 (15)	144

Symmetry codes: (i) 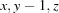; (ii) 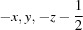.

**Table 2:** Experimental details

Crystal data	
Chemical formula	C_18_H_18_O_6_
*M*_r_	330.32
Crystal system, space group	Monoclinic, *C*2/*c*
Temperature (K)	105
*a*, *b*, *c* (Å)	28.5800 (14), 4.0632 (2), 14.4806 (7)
β (°)	115.100 (1)
*V* (Å^3^)	1522.78 (13)
*Z*	4
Radiation type	Mo *K*α
μ (mm^−1^)	0.11
Crystal size (mm)	0.56 × 0.29 × 0.22

Data collection	
Diffractometer	Bruker PHOTON CCD
Absorption correction	Multi-scan (*SADABS*; Bruker, 2007 ▸)
*T*_min_, *T*_max_	0.602, 0.746
No. of measured, independent and observed [*I* > 2σ(*I*)] reflections	17902, 2053, 1819
*R*_int_	0.037
(sin θ/λ)_max_ (Å^−1^)	0.685

Refinement	
*R*[*F* ^2^ > 2σ(*F* ^2^)], *wR*(*F* ^2^), *S*	0.038, 0.109, 1.10
No. of reflections	2053
No. of parameters	111
H-atom treatment	H-atom parameters constrained
Δρ_max_, Δρ_min_ (e Å^−3^)	0.41, −0.24

Computer programs: *APEX2* and *SAINT* (Bruker, 2007 ▸), *SHELXS97* (Sheldrick, 2008 ▸), *SHELXL2014* (Sheldrick, 2015 ▸), *WinGX* (Farrugia, 2012 ▸), *DIAMOND* (Brandenburg, 2004 ▸), *ChemBioDraw* (Cambridge Soft, 2009 ▸) and *publCIF* (Westrip, 2010 ▸).

## supplementary crystallographic information

### Crystal data

**Table:**

C_18_H_18_O_6_	*F*(000) = 696
*M**_r_* = 330.32	*D*_x_ = 1.441 Mg m^−^^3^
Monoclinic, *C*2/*c*	Mo *K*α radiation, λ = 0.71073 Å
*a* = 28.5800 (14) Å	Cell parameters from 9942 reflections
*b* = 4.0632 (2) Å	θ = 2.8–29.1°
*c* = 14.4806 (7) Å	µ = 0.11 mm^−^^1^
β = 115.100 (1)°	*T* = 105 K
*V* = 1522.78 (13) Å^3^	Needle, colourless
*Z* = 4	0.56 × 0.29 × 0.22 mm

### Data collection

**Table:**

Bruker PHOTON CCD diffractometer	2053 independent reflections
Radiation source: fine-focus sealed tube	1819 reflections with *I* > 2σ(*I*)
Graphite monochromator	*R*_int_ = 0.037
ω scans	θ_max_ = 29.2°, θ_min_ = 2.8°
Absorption correction: multi-scan (*SADABS*; Bruker, 2007)	*h* = −38→38
*T*_min_ = 0.602, *T*_max_ = 0.746	*k* = −5→5
17902 measured reflections	*l* = −19→19

### Refinement

**Table:**

Refinement on *F*^2^	Primary atom site location: structure-invariant direct methods
Least-squares matrix: full	Secondary atom site location: difference Fourier map
*R*[*F*^2^ > 2σ(*F*^2^)] = 0.038	Hydrogen site location: inferred from neighbouring sites
*wR*(*F*^2^) = 0.109	H-atom parameters constrained
*S* = 1.10	*w* = 1/[σ^2^(*F*_o_^2^) + (0.0534*P*)^2^ + 1.1903*P*] where *P* = (*F*_o_^2^ + 2*F*_c_^2^)/3
2053 reflections	(Δ/σ)_max_ < 0.001
111 parameters	Δρ_max_ = 0.41 e Å^−^^3^
0 restraints	Δρ_min_ = −0.24 e Å^−^^3^

### Special details

**Table:**

Geometry. All e.s.d.'s (except the e.s.d. in the dihedral angle between two l.s. planes) are estimated using the full covariance matrix. The cell e.s.d.'s are taken into account individually in the estimation of e.s.d.'s in distances, angles and torsion angles; correlations between e.s.d.'s in cell parameters are only used when they are defined by crystal symmetry. An approximate (isotropic) treatment of cell e.s.d.'s is used for estimating e.s.d.'s involving l.s. planes.

### Fractional atomic coordinates and isotropic or equivalent isotropic displacement parameters (Å^2^)

**Table:**

	*x*	*y*	*z*	*U*_iso_*/*U*_eq_	
C1	0.22923 (4)	0.2306 (3)	0.01755 (7)	0.0163 (2)	
C2	0.17986 (4)	0.3583 (3)	−0.03782 (7)	0.0175 (2)	
H2	0.1730	0.4703	−0.0998	0.021*	
C3	0.14011 (4)	0.3300 (3)	−0.00694 (7)	0.0170 (2)	
C4	0.14924 (4)	0.1625 (3)	0.08424 (8)	0.0170 (2)	
C5	0.19832 (4)	0.0299 (3)	0.14041 (8)	0.0193 (2)	
H5	0.2052	−0.0851	0.2020	0.023*	
C6	0.23708 (4)	0.0637 (3)	0.10753 (8)	0.0193 (2)	
H6	0.2701	−0.0293	0.1473	0.023*	
C7	0.09039 (4)	0.4858 (3)	−0.07720 (8)	0.0192 (2)	
C8	0.01027 (4)	0.7077 (3)	−0.10223 (9)	0.0256 (3)	
H8A	−0.0111	0.7454	−0.0653	0.038*	
H8B	−0.0086	0.5685	−0.1618	0.038*	
H8C	0.0184	0.9191	−0.1246	0.038*	
C9	0.11816 (5)	−0.0599 (3)	0.20034 (8)	0.0229 (2)	
H9A	0.0868	−0.0586	0.2121	0.034*	
H9B	0.1470	0.0309	0.2601	0.034*	
H9C	0.1262	−0.2863	0.1888	0.034*	
O1	0.10999 (3)	0.1361 (2)	0.11283 (6)	0.02064 (19)	
O2	0.05778 (3)	0.5450 (2)	−0.03563 (6)	0.0232 (2)	
O3	0.08127 (3)	0.5572 (3)	−0.16406 (7)	0.0354 (3)	

### Atomic displacement parameters (Å^2^)

**Table:**

	*U*^11^	*U*^22^	*U*^33^	*U*^12^	*U*^13^	*U*^23^
C1	0.0173 (5)	0.0161 (5)	0.0141 (4)	−0.0009 (4)	0.0053 (4)	−0.0023 (4)
C2	0.0182 (5)	0.0194 (5)	0.0133 (4)	−0.0012 (4)	0.0051 (4)	−0.0004 (4)
C3	0.0162 (4)	0.0181 (5)	0.0142 (4)	−0.0010 (4)	0.0040 (4)	−0.0018 (4)
C4	0.0187 (5)	0.0168 (5)	0.0156 (4)	−0.0020 (4)	0.0072 (4)	−0.0027 (4)
C5	0.0216 (5)	0.0201 (5)	0.0153 (4)	0.0013 (4)	0.0071 (4)	0.0023 (4)
C6	0.0186 (5)	0.0208 (5)	0.0164 (5)	0.0025 (4)	0.0054 (4)	0.0008 (4)
C7	0.0165 (5)	0.0223 (5)	0.0170 (5)	−0.0015 (4)	0.0055 (4)	0.0001 (4)
C8	0.0185 (5)	0.0324 (6)	0.0240 (5)	0.0064 (5)	0.0073 (4)	0.0036 (5)
C9	0.0288 (6)	0.0234 (6)	0.0203 (5)	0.0022 (4)	0.0141 (4)	0.0036 (4)
O1	0.0207 (4)	0.0247 (4)	0.0184 (4)	0.0013 (3)	0.0101 (3)	0.0038 (3)
O2	0.0193 (4)	0.0323 (5)	0.0172 (4)	0.0060 (3)	0.0068 (3)	0.0015 (3)
O3	0.0223 (4)	0.0622 (7)	0.0219 (4)	0.0115 (4)	0.0096 (3)	0.0158 (4)

### Geometric parameters (Å, º)

**Table:**

C1—C2	1.3940 (14)	C6—H6	0.9500
C1—C6	1.3998 (14)	C7—O3	1.2067 (14)
C1—C1^i^	1.4846 (19)	C7—O2	1.3285 (13)
C2—C3	1.3909 (14)	C8—O2	1.4490 (13)
C2—H2	0.9500	C8—H8A	0.9800
C3—C4	1.4080 (14)	C8—H8B	0.9800
C3—C7	1.4928 (14)	C8—H8C	0.9800
C4—O1	1.3549 (12)	C9—O1	1.4293 (13)
C4—C5	1.3971 (15)	C9—H9A	0.9800
C5—C6	1.3859 (15)	C9—H9B	0.9800
C5—H5	0.9500	C9—H9C	0.9800
			
C2—C1—C6	116.04 (9)	O3—C7—O2	123.09 (10)
C2—C1—C1^i^	121.65 (11)	O3—C7—C3	122.32 (10)
C6—C1—C1^i^	122.31 (11)	O2—C7—C3	114.58 (9)
C3—C2—C1	123.36 (9)	O2—C8—H8A	109.5
C3—C2—H2	118.3	O2—C8—H8B	109.5
C1—C2—H2	118.3	H8A—C8—H8B	109.5
C2—C3—C4	119.32 (9)	O2—C8—H8C	109.5
C2—C3—C7	114.66 (9)	H8A—C8—H8C	109.5
C4—C3—C7	126.03 (9)	H8B—C8—H8C	109.5
O1—C4—C5	123.27 (9)	O1—C9—H9A	109.5
O1—C4—C3	118.48 (9)	O1—C9—H9B	109.5
C5—C4—C3	118.25 (10)	H9A—C9—H9B	109.5
C6—C5—C4	120.87 (10)	O1—C9—H9C	109.5
C6—C5—H5	119.6	H9A—C9—H9C	109.5
C4—C5—H5	119.6	H9B—C9—H9C	109.5
C5—C6—C1	122.15 (10)	C4—O1—C9	118.03 (8)
C5—C6—H6	118.9	C7—O2—C8	114.87 (9)
C1—C6—H6	118.9		
			
C6—C1—C2—C3	0.89 (16)	C2—C1—C6—C5	−0.64 (16)
C1^i^—C1—C2—C3	−179.29 (11)	C1^i^—C1—C6—C5	179.55 (12)
C1—C2—C3—C4	−0.55 (16)	C2—C3—C7—O3	17.33 (16)
C1—C2—C3—C7	179.71 (10)	C4—C3—C7—O3	−162.39 (12)
C2—C3—C4—O1	−179.68 (9)	C2—C3—C7—O2	−161.30 (10)
C7—C3—C4—O1	0.03 (16)	C4—C3—C7—O2	18.97 (16)
C2—C3—C4—C5	−0.08 (15)	C5—C4—O1—C9	−5.22 (15)
C7—C3—C4—C5	179.63 (10)	C3—C4—O1—C9	174.36 (10)
O1—C4—C5—C6	179.90 (10)	O3—C7—O2—C8	−0.89 (17)
C3—C4—C5—C6	0.32 (16)	C3—C7—O2—C8	177.74 (10)
C4—C5—C6—C1	0.05 (17)	C2—C1—C1^i^—C6^i^	0.2 (2)

Symmetry code: (i) −*x*+1/2, −*y*+1/2, −*z*.

### Hydrogen-bond geometry (Å, º)

**Table:**

*D*—H···*A*	*D*—H	H···*A*	*D*···*A*	*D*—H···*A*
C9—H9*C*···O1^ii^	0.98	2.55	3.4759 (15)	158
C8—H8*B*···O3^iii^	0.98	2.50	3.3407 (15)	144

Symmetry codes: (ii) *x*, *y*−1, *z*; (iii) −*x*, *y*, −*z*−1/2.
